# Factors associated with the morphology of the mandibular symphysis and soft tissue chin

**DOI:** 10.1590/2177-6709.26.4.e2119347.oar

**Published:** 2021-09-10

**Authors:** Karine EVANGELISTA, Maria Alves Garcia Santos SILVA, David NORMANDO, José VALLADARES-NETO

**Affiliations:** 1 Universidade Federal de Goiás, Faculdade de Odontologia, Departamento de Ortodontia (Goiânia/GO, Brazil).; 2 Universidade Federal de Goiás, Faculdade de Odontologia, Departamento de Ciências Estomatológicas (Goiânia/GO, Brazil).; 3 Universidade Federal do Pará, Faculdade de Odontologia, Departamento de Ortodontia (Belém/PA, Brazil).

**Keywords:** Chin, Symphysis, Morphology, Adult, Genioplasty

## Abstract

**Objectives:**

This study aimed to (I) assess the morphology of the symphysis and soft tissue chin associated with sex, age and sagittal/vertical skeletal patterns, and (II) identify the individual and combined contributions of these variables to different portions of the symphysis.

**Methods:**

This cross-sectional study included 195 lateral cephalometric radiographs from untreated adults. Alveolar, basal, and soft tissue of the symphysis were measured by an X/Y cranial base coordinate system, and divided in accordance to four predictor variables: sex, age, and sagittal/vertical skeletal patterns. Parametric tests were conducted for comparison and correlation purposes, while multiple regression analysis was performed to explore combined interactions.

**Results:**

Alveolar inclination is related to sagittal and vertical patterns, and both explained 71.4% of the variations. Alveolar thickness is weakly predicted and poorly influenced by age. Symphysis height was 10% higher in males, and associated with a vertical skeletal pattern and sex, and both explained 43.6% of variations. Basal symphyseal shows an individual thickness, is larger in males, and vertically short-positioned with age. Soft tissue chin is not necessarily related to the size of the underling skeletal pattern, and enlarges with age, even in adulthood.

**Conclusions:**

The symphysis and surrounding tissues are influenced by sex, age, and sagittal and vertical patterns, acting differently on the alveolar, basal and soft tissue portions. Sagittal and vertical skeletal patterns are the strongest association on alveolar symphysis inclination, whereas sex and age acts on the vertical symphysis position and soft tissues thickness.

## RESUMO

**Introdução:**

O presente estudo objetivou: 1) avaliar a morfologia da sínfise mandibular e dos tecidos tegumentares do mento, associada ao sexo, idade, padrões sagital e vertical; e 2) identificar as contribuições dessas variáveis, individualmente ou combinadas, às diferentes regiões da sínfise.

**Métodos:**

Este estudo transversal incluiu 195 radiografias cefalométricas laterais de adultos não tratados ortodonticamente. Os tecidos alveolar, basal e mole da sínfise foram medidos por um sistema de coordenadas x, y e z da base do crânio e divididos de acordo com quatro variáveis preditoras: sexo, idade e padrões esqueléticos sagitais e verticais. Testes paramétricos foram conduzidos para fins de comparação e correlação, enquanto a análise de regressão múltipla foi realizada para explorar as interações combinadas.

**Resultados:**

A inclinação alveolar está relacionada aos padrões sagitais e verticais, e ambos explicaram 71,4% das variações. A espessura alveolar é fracamente prevista e pouco influenciada pela idade. A altura da sínfise foi 10% maior no sexo masculino e esteve associada ao padrão esquelético vertical e ao sexo, sendo que ambos explicaram 43,6% das variações. A sínfise basal mostra espessura individual, é maior no sexo masculino e verticalmente curta com a idade. O tecido mole do mento não está necessariamente relacionado ao tamanho do padrão esquelético subjacente e aumenta com a idade, mesmo na idade adulta.

**Conclusões:**

A sínfise e os tecidos circundantes são influenciados pelo sexo, idade e padrões sagitais e verticais, que atuam de forma diferenciada nas porções alveolar, basal e de tecidos tegumentares. Os padrões esqueléticos sagitais e verticais são a associação mais forte na inclinação da sínfise alveolar, enquanto o sexo e a idade atuam na posição vertical da sínfise e na espessura dos tecidos tegumentares.

## INTRODUCTION

The mandibular symphysis is the anatomical anterior part of the mandible composed of cortical and alveolar bones. Differently, the chin, or mentum, is the projected part of the mandibular symphysis, constituting a feature unique to modern humans.^1^ In turn, the adjacent soft tissue below the lower lip is called soft tissue chin. The symphysis and adjacent tissues make up an interactive and complex anatomical structure, didactically divided into three portions: two hard tissues and one soft tissue. The hard tissues constitute the alveolar and basal portions. The alveolar ridge accommodates the mandibular incisors, and its inclination usually matches the long axis of the alveolar symphysis.^2^ The basal portion constitutes the mandibular symphysis itself, with a more apical location when compared to the alveolar portion.^3^ On the other hand, the soft tissue chin represents the integumental mentum, which is supported and designed by the underlying basal symphysis, dentoalveolar projection, and soft tissue thickness.^4^

Identifying the factors associated with the morphology of the symphysis and adjacent structures can be useful for basic and applied sciences. Alveolar symphyseal inclination can be affected by anteroposterior orthodontic movement in compensatory or decompensatory treatments for skeletal discrepancies,^5^ and also in arch-perimeter changes for tooth-size discrepancies.^6^ This aspect has been a concern when considering a safe anteroposterior movement of the mandibular incisors, preventing periodontal damage such as bone fenestration and dehiscence.^7^ The inclination of the long axis of the basal symphysis is one of the characteristics used to predict mandibular rotation and projection during growth.^8^ This symphyseal site can also provide autogenous bone for bone grafting prior to dental implant placement.^9^ Furthermore, both bone and chin soft tissue play crucial roles in facial aesthetics, and are therefore vital when making surgical case decisions in cases of genioplasty.^10^

Previous cross-sectional^3^ ^,^ ^4^ ^,^ ^11^ ^-^ ^17^ and longitudinal^18^ ^-^ ^20^ studies have shown that the morphology and position of the symphysis and adjacent structures can be influenced by age, sex, and sagittal/vertical skeletal patterns. Inconsistent results were found in some of these studies, probably because unclear information is available about the influence of each of these factors on the symphysis morphology. Despite some studies^12^ ^,^ ^17^ have used a multivariate statistic for data analysis, age was a factor not considered. In addition, proper chin position is a relevant goal of orthodontic treatment, and understanding the influencing factors is encouraging.

The present study hypothesized that different regions of the symphysis and adjacent tissues are influenced individually or in association by different predictor variables. Thus, the aim of this study was to assess the contributions of sex, age, and sagittal/vertical patterns to the morphology variation of the alveolar, basal, and soft tissue portions of the symphysis and surrounding tissues, using a multiple regression model. 

## MATERIAL AND METHODS

This cross-sectional cephalometric study was approved by the Institutional Review Board/Federal University of Uberlândia (Uberlândia/MG, Brazil), with the protocol number 247/07. The STROBE guidelines for observational studies were followed.^21^

### SAMPLE

A sample of 195 lateral cephalometric radiographs of untreated white adults (100 males and 95 females) was consecutively selected from the pretreatment orthodontic records of 563 patients. The sample presented varying degrees of skeletal severity, and was adjusted to balance the number of subjects with anteroposterior and vertical skeletal patterns.

The following inclusion criteria were followed: adult patients (males > 18y and females > 16y); facial symmetry (detected by facial photographs); presence of all teeth, except third molars; and good quality digital lateral cephalometric radiographs, teeth in maximum intercuspal position, lips at rest and a natural head position. According to the exclusion criteria, radiographic images presenting advanced periodontal disease; signs of facial or dental trauma; syndromes or congenital craniofacial anomalies, such as cleft lip or palate; and previous orthodontic, prosthetic, or surgical procedures, were not considered. 

The total sample was divided according to the predictor variables sex, age, and sagittal and vertical skeletal patterns. Age division was distributed as follows: younger (< 25 years old, n* *= 89); middle (≥ 25 and < 35 years old, n* *= 49), and older (≥ 35 years old, n* *= 57). The sagittal pattern was divided according to: skeletal Class I (well-balanced face, with ANB angle between 0° and 4°, n* *= 60); skeletal Class II malocclusion (ANB > 4°, n* *= 64), and skeletal Class III malocclusion (ANB ˂ 0°, n* *= 71). The vertical pattern was divided according to the mandibular plane angle, with normodivergent (> 28 SN.GoGn ˂ 34°, n* *= 62), hyperdivergent (SN.GoGn ≥ 34°, n* *= 67) and hypodivergent (SN.GoGn ˂ 28°, n* *= 66). Table 1 describes the sample characteristics.

**Table 1:** Sample characteristics (n = 195).

		Horizontal pattern				Vertical pattern				Age			
		Class I	Class II	Class III	n	Low MP-angle	Medium MP-angle	High MP-angle	n	< 25	25,0 - 34,9	≥ 35	n
n		60	64	71	195	66	62	67	195	89	49	57	195
Sex (frequency)	Male	30	30	40	100	37	31	32	100	53	20	25	98
	Female	30	34	31	95	29	31	35	95	36	29	32	97
Age (years)	Mean (SD)	24.0 (6.2)	3.2 (9.1)	30.7 (11.1)		28.8 (10.4)	29.1 (9.1)	28.7 (9.4)		20.6 (2.5)	28.8 (2.5)	41.3 (6.1)	
	Min./ Max.	16.4/ 42.7	16.0/ 55.0	16.0/ 63.0		16.0/ 63.0	16.0/ 53.0	16.4/ 55.0		16.0/ 24.0	25.0/ 34.0	35.0/ 63.0	
MP- angle	Mean (SD)	30.0 (5.8)	31.5 (7.6)	30.0 (5.6)		23.7 (3.1)	30.2 (1.7)	37.5 (3.1)		29.9 (6.1)	31.1 (5.4)	30.7 (7.3)	
	Min/ Max	16.0/ 43.5	14.0/ 46.0	17.0/ 42.0		14.0/ 28.0	28.0/ 33.0	34.0/ 46.0		14.0/ 46.0	17.0/ 42.0	16.0/ 44.5	
ANB (angle)	Mean (SD)	2.3 (1.2)	6.8 (1.6)	-3.3 (2.3)		-0.9 (4.7)	1.7 (4.6)	2.6 (4.4)		1.5 (4.2)	2.3 (4.7)	1.5 (5.11)	
	Min/ Max	0.0/ 4.0	4.5/ 12.0	-11.0/ -0.5		-11.0/ 9.0	-6.0/ 12.0	-8.0/ 9.0		-11.0/ 10.0	-6.0/ 12.0	-8.0/ 10.0	

### CEPHALOMETRIC ASSESSMENT

**Figure 1:**
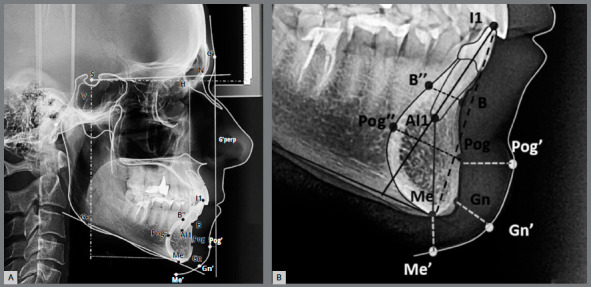
Landmarks, lines, and planes. S (midpoint of sella turcica), A (most concave point of anterior maxilla), N (most anterior point on frontonasal suture), B (most concave point on mandibular symphysis), B’’ (most posterior point on alveolar mandibular symphysis projected by a parallel line to MP passing through point B), Go (most inferior point on mandible angle), Gn (most anteroinferior point on mandibular symphysis), Gn’ (gnathion soft tissue), G’ (glabella soft tissue), I1 (Incisor edge of the mandibular incisor), Al1 (root apex of the mandibular incisor), Me (lowest point on mandibular symphysis), Me’ (soft tissue menton), Pog (most anterior point of mandibular symphysis), Pog’ (soft tissue pogonion), and Pog’’ (most posterior point of basal mandibular symphysis projected by a parallel line to MP passing through Pog point). S-N (line connecting S and N), H (sagittal line from the S at 7° inferior to the original S-N line), V (line from S perpendicular to H), MP (mandibular plane, line connecting Go and Me), G’-perp (line from G´ perpendicular to H line), and L1 (line connecting incisal edge and root apex).

Cephalograms were traced by hand on acetate paper in a darkened room, by an experienced and calibrated orthodontist. Eleven angular and linear measurements were obtained based on sixteen cephalometric landmarks (Fig 1 and Table 2). Variables were grouped in accordance with the alveolar, basal and soft tissue portions of the symphysis and surrounding tissues. Digital cephalometric radiographs were obtained with standardized settings (90 kV, 12.6 mA).

**Table 2:** Cephalometric measurements.

Variable	Identification	Type	Definition
Alveolar symphysis			
Inclination	IMPA	Angular (degrees)	Angulation between the long axis of mandibular incisor (L1-AL1) and the mandibular plane (MP).
Thickness	B-B’’	Linear (mm)	Linear distance between points B and B’’.
Height	I1-Me	Linear (mm)	Linear distance between points I1 and Me, representing the alveolar and basal heights, and including the mandibular incisor.
Basal symphysis			
Inclination	AL1Me.MP	Angular (degrees)	Angulation between the height of basal symphysis (AL1-Me) and the mandibular plane (MP).
Thickness	Pog-Pog’’	Linear (mm)	Linear distance between points Pog and Pog’’.
Horizontal position	V-Gn	Linear (mm)	Minor linear distance between line V and point Gn, representing the sagittal position of the symphysis on the face.
Vertical position	H-Gn	Linear (mm)	Minor linear distance between line H and point Gn, representing the vertical position of the symphysis on the face.
Soft tissue chin			
Projection in relation to Glabella	G’perp-Pog’	Linear (mm)	Minor linear distance between line G’perp and point Pog’, representing the sagittal projection of the chin.
Thickness (Pog)	Pog-Pog’	Linear (mm)	Minor linear distance between points Pog and Pog’, representing the anterior thickness of the chin soft tissue.
Thickness (Gn)	Gn-Gn’	Linear (mm)	Minor linear distance between points Gn and Gn’, representing the more anteroinferior thickness of the chin soft tissue.
Thickness (Me)	Me-Me’	Linear (mm)	Minor linear distance between points Me and Me’, representing the inferior thickness of the chin soft tissue.

### STATISTICAL ANALYSIS

A *post-hoc* power analysis was undertaken using G*Power software version 3.2.9.2. (Germany)^22^. Analysis was based on total sample size and number of tested predictors, by using multiple linear regression model of analysis, with fixed effects (alpha = 0.05; effect size = 0.09). Sample power was 0.93. To assess the reliability of the method, 80 lateral cephalometric radiographs were randomly selected and re-measured by the same researcher with an interval of at least two weeks. The intra-class correlation coefficient (ICC) at *p*<0.05 was used to determine intra-examiner reliability followed by Bland-Altman plots analysis.

The primary outcome variables (Y) were: alveolar, basal, and soft tissue portion characteristics. The predictor variables (X) were: sex, age, sagittal and vertical skeletal patterns. Data were analyzed using comparative statistics for age, sagittal and vertical patterns (One-way ANOVA and Kruskal-Wallis tests, followed by Tukey test and pairwise comparison analysis, respectively). Sex groups were evaluated using *t* test. Pearson correlation analysis was performed to identify statistically significant variables. Multiple linear regression was undertaken using multivariate general linear models, adjusting for potential confounders, and in order to evaluate interaction between the independent factors and each predictor variable. 2-tailed statistical significance was set at *p*˂0.05, carried out using the software SPSS 23.0 for Windows (IBM Corp, Armonk, NY). 

## RESULTS

### RELIABILITY

**Figure 2:**
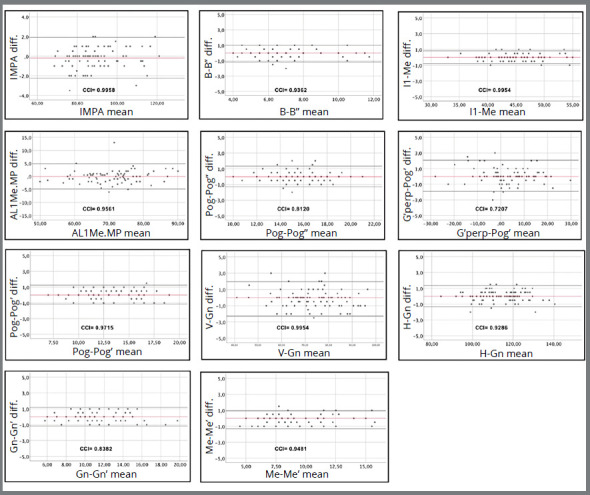
Bland-Altman plots with the mean difference of measurements, 95% CI and the CCI values of each variable.

Figure 2 shows all cephalometric measurements with CCI values and respective Bland-Altman plots. The results revealed a high intra-examiner agreement for most variables. The G’perp-Pog’ (CCI=0.7207) was an exception, probably due to limitations in the geometric arrangement and distance in measuring. The cephalometric method used in this study is a reliable tool to evaluate the morphology of the symphysis and adjacent tissues.

### SAMPLE CHARACTERISTICS

Sample characteristics showed similar distributions of sex, and sagittal and vertical discrepancies (Table 1).

### UNIVARIATE ANALYSIS

The alveolar inclination was similar for both genders, although influenced by the sagittal and vertical patterns. It was anteriorly inclined in Class II and sagittal cases, and posteriorly inclined in Class III and vertical cases. Alveolar symphyseal thickness was not influenced by age or sex, and was thinner in individuals with a Class III skeletal combined with a hyperdivergent pattern. Symphyseal height was longer in the vertical pattern and 10% higher in males. Basal symphyseal inclination was less influenced by the sagittal pattern. Basal symphyseal thickness was greater in males and not associated with any other variable. The sagittal and vertical position was influenced by the sagittal and vertical skeletal patterns, and vertically reduced with age. Projection of the chin’s soft tissue was associated with the sagittal and vertical patterns and was more projected in Class III and less projected in a high mandibular plane angle. A difference between younger and older adults was also detected in the chin soft tissue. With age, a mean increase of 1.3 mm in younger and 1.7 mm in older adults could be found for Pog’, Gn’ and Me’ regions (Table 3).

**Table 3:** Alveolar, basal, and soft tissue in different sagittal and vertical patterns, age, and sex groups.

	Sagittal pattern (n=195)			Vertical pattern (n=195)			Age (years) (n=193)			Sex (n=195)		
	Class I (n=60)	Class II (n=64)	Class III (n=71)	Low MP (n=66)	Medium MP (n=62)	High MP (n=67)	>25 (n=89)	25-34,9 (n=49)	≥35 (n=50)	Male (n=100)	Female (n=95)	P- value
Alveolar symphysis												
IMPA (degrees)	95.0 (6.4)^A^	103.9 (8.9)^B^	83.8 (7.6)^C^	97.0 (12.0)^A^	94.3 (11.8)^B^	90.0 (9.4)^C^	95.8 (11.07)^A^	93.4 (12.7) ^A^	92.4 (11.6) ^A^	94.04 (11.5)	93.7 (11.3)	0.849
B-B” (mm)	6.9 (1.0)^A^	7.1 (1.7)^A^	5.9 (1.2)^B^	7.2 (1.5)^A^	6.6 (1.4)^B^	5.9 (0.9)^C^	6.7 (1.4)^A^	6.7 (1.4)^A^	6.2 (1.4)^A^	6.8 (1.5)	6.4 (1.2)	0.219
I1-Me (mm)	41.8 (3.2)^A^	42.8 (4.2)^A^	42.1 (3.8)^A^	40.7 (3.1)^A^	42.1 (3.6)^B^	44.0 (3.7)^C^	41.6 (4.0)^A^	42.1 (3.4)^A^	42.1 (3.9)^A^	44.1 (3.5)	40.3 (3.0)	< 0.001
Basal symphysis												
A1Me.MP (degrees)	71.3 (4.8)^A^	74.8 (8.8)^B^	69.7 (8.2)^A^	72.9 (7.1)^A^	72.6 (9.2)^A^	70.1 (6.9)^A^	72.3 (8.1)^A^	71.4 (7.8)^A^	72.2 (8.6)^A^	71.2 (8.0)	72.5 (7.6)	0.178
Pog-Pog’’ (mm)	14.1 (2.0)^A^	14.8 (2.2)^A^	14.3 (1.8)^A^	14.7 (2.2)^A^	14.6 (2.1)^A^	14.0 (1.2)^A^	14.4 (1.9)^A^	14.6 (1.8)^A^	14.3 (2.2)^A^	14.9 (1.9)	13.9 (2.0)	< 0.001
V-Gn (mm)	62.7 (8.1)^A^	56.4 (9.4)^B^	72.3 (9.2)^C^	71.5 (9.0)^A^	64.1 (9.2)^B^	56.9 (10.1)^C^	63.7 (9.3)^A^	63.9 (11.8)^A^	65.0 (12.2)^A^	67.2 (10.5)	61.0 (11.0)	< 0.001
H-Gn (mm)	108.1 (8.2)^A^	105.5 (14.1)^A^	108.5 (9.5)^A^	102.4 (7.8)^A^	108.4 (14.0)^B^	111.4 (8.4)^C^	108.4 (15.5)^A^	107.0 (8.2)^AB^	105.1 (9.7)^B^	111.4 (12.2)	103.1 (7.3)	< 0.001
Soft tissue chin												
G’perp-Pog’ (mm)	-3.2 (7.1)^A^	-8.6 (7.8)^B^	7.1 (7.5)^C^	4.4 (9.3)^A^	-0.9 (9.1)^B^	-7.0 (8.1)^C^	-1.5 (9.1)^A^	-1.1 (10.8)^A^	0.2 (10.7)^A^	-0.8 (9.8)	-1.6 (10.3)	0.480
Pog-Pog’ (mm)	11.8 (2.1)^A^	12.8 (2.5)^B^	12.4 (2.5)^AB^	12.5 (2.8)^A^	12.3 (2.4)^A^	12.3 (2.00)^A^	12.0 (2.3)^A^	11.8 (2.4)^AB^	13.3 (2.3)^B^	12.9 (2.6)	11.8 (2.0)	0.002
Gn-Gn’ (mm)	9.6 (2.4)^A^	10.5 (2.8)^A^	10.6 (2.9)^A^	11.0 (3.0)^A^	10.4 (2.6)^A^	9.4 (2.4)^B^	8.9 (2.3)^A^	9.6 (2.0)^A^	11.6 (2.8)^B^	10.8 (2.8)	9.7 (2.5)	0.006
Me-Me’ (mm)	8.3 (2.0)^A^	8.4 (2.5)^A^	9.0 (2.3)^A^	8.5 (2.6)^A^	8.7 (2.1)^A^	8.4 (2.1)^A^	8.3 (2.0)^A^	8.1 (2.1)^A^	9.1 (2.4)^B^	9.3 (2.5)	7.8 (1.7)	<0.001

One-way ANOVA and t tests. Different letters represent statistical significance among groups and same letters, no statistical significance.

### CORRELATION AND MULTIPLE REGRESSION ANALYSIS

Tables 4 and 5 present the correlation and multiple regression analysis, respectively. Dentoalveolar symphyseal morphology exerted different influences in terms of inclination, thickness and total height. The main factor affecting alveolar inclination was sagittal discrepancy (*r*= 0.567*)*, and the combination of sagittal and vertical patterns explained 71.1% of inclination variability. Alveolar thickness was explained in part by the vertical and sagittal patterns *(R* ^*2*^ **=* *0.279, *P*=* *<0.001*)*. Regarding symphyseal height, the main contributing factors were sex (*r*=* *0.243) and vertical patterns (*r*=* *0.149), highlighting these factors in this measurement variability (*R* ^*2*^ *=*0.436).

**Table 4:** Outcome variables (alveolar, basal, and soft tissue) correlated with predictor variables (horizontal and vertical discrepancies, sex, and age).

	ANB (Sagittal discrepancy)			MP-angle (Vertical discrepancy)			Sex^a^			Age (years)		
	Β value (CI 95%)	r	P- value	Β value (CI 95%)	r	P- value	Β value (CI 95%)	r	P- value	Β value (CI 95%)	r	P- value
Alveolar symphysis												
IMPA	1.85 (1.62/2.08)	0.567	< 0.001**	-0.47 (-0.72/ -0.23)	0.07	<0.001**	0.31 (-2.92/3.55)	0	0.849	-0.13 (-0.14/0.12)	0	0.853
B-B’’	0.08 (0.04/0.12)	0.079	< 0.001**	-0.09 (-0.12/-0.06)	0.166	<0.001**	0.36 (-0.03/0.77)	0.017	0.073	-0.007 (-0.02/0.01)	0.003	0.439
I1-Me	0.07 (-0.04/0.19)	0.009	0.199	0.22 (0.15/0.30)	0.149	<0.001**	3.71 (2.78/4.64)	0.243	<0.001**	-0.006 (-0.05/0.03)	0	0.809
Basal symphysis												
A1Me.MP	0.51 (0.28/0.74)	0.092	< 0.001**	-0.24 (-0.41/-0.07)	0.04	0.005**	-1.2 (-3.5/0.93)	0.007	0.255	0.02 (-0.06/0.11)	0.002	0.585
Pog-Pog’’	0.02 (-0.03/0.08)	0.003	0.447	-0.05 (-0.10/-0.01)	0.033	0.01**	1.02 (0.46/1.59)	0.063	<0.001**	0.001 (-0.02/0.02)	0	0.943
V-Gn	-1.54 (-1.80/-1.28)	0.41	< 0.001**	-1.03 (-1.23/-0.83)	0.351	<0.001**	6.19 (3.15/9.22)	0.077	<0.001**	0.00 (-0.13/0.13)	0	0.997
H-Gn	-0.17 (-0.50/0.16)	0.005	0.313	0.59 (0.36/0.82)	0.12	0.001**	8.26 (5.40/11.13)	0.143	<0.001**	-0.11 (-0.24/0.01)	0.016	0.07
Soft tissue chin												
G’perp-Pog’	-1.51 (-1.73/-1.29)	0.489	< 0.001**	-0.84 (-1.03/-0.65)	0.285	<0.001**	0.77 (-2.06/3.62)	0.001	0.591	0.02 (-0.09/0.14)	0.001	0.670
Pog-Pog’	0.03 (-0.04/0.10)	0.004	0.368	-0.02 (-0.07/0.03)	0.003	0.467	1.09 (0.42/1.76)	0.051	0.001**	0.03 (0.008/0.06)	0.032	0.013*
Gn-Gn’	-0.02 (-0.11/0.05)	0.002	0.541	-0.11 (-0.17/-0.53)	0.068	<0.001**	1.11 (0.35/1.88)	0.041	0.004**	0.06 (0.02/0.09)	0.068	<0.001**
Me-Me’	-0.04 (-0.11/0.02)	0.007	0.246	0.01 (-0.04/0.06)	0.001	0.700	1.51 (0.90/2.12)	0.111	<0.001**	0.02 (-0.003/0.05)	0.016	0.08

Pearson correlation analysis.

^a^ Female = 1 and male = 2; CI= confidence interval, P < 0.05 ** P < 0.01.

**Table 5:** Interaction models of horizontal pattern (ANB), vertical pattern (MP Angle), sex, and age using multiple regression model for predicting alveolar, basal and soft tissue.

	ANB vs MP Angle		ANB vs Sex		ANB vs Age		MP Angle vs Sex		MP Angle vs Age		Sex vs Age		ANB vs MP Angle vs Sex vs age	
Measurements	R^2^	P	R^2^	P	R^2^	P	R^2^	P	R^2^	P	R^2^	P	R^2^	P
Alveolar symphysis														
IMPA	0.711	<0.001***	0.566	<0.001***	0.563	<0.001***	0.061	0.001**	0.061	0.001**	0.10	0.966	0.709	<0.001***
B-B’’	0.279	<0.001***	0.091	<0.001***	0.073	<0.001***	0.164	0.007**	0.160	0.017**	0.002	0.456	0.283	<0.001***
I1-Me	0.142	<0.001***	0.250	<0.001***	0.001	0.426	0.436	<0.001***	0.141	<0.001***	0.235	0.152	0.434	<0.001***
Basal symphysis														
A1Me.MP	0.144	<0.001***	0.086	<0.001***	0.084	<0.001***	0.041	<0.001***	0.032	0.038*	0.009	0.002**	0.146	<0.001***
Pog-Pog’’	0.030	0.019*	0.058	0.001**	0.007	0.747	0.078	<0.001***	0.024	<0.001***	0.054	<0.001***	0.077	0.001**
V-Gn	0.658	<0.001***	0.464	<0.001***	0.404	<0.001***	0.389	<0.001***	0.345	<0.001***	0.068	<0.001***	0.692	<0.001***
H-Gn	0.127	<0.001***	0.137	<0.001***	0.011	0.127	0.290	<0.001***	0.129	<0.001***	0.149	<0.001***	0.312	<0.001***
Soft tissue chin														
G´perp-Pog’	0.673	<0.001***	0.483	<0.001***	0.484	<0.001***	0.278	<0.001***	0.279	<0.001***	0.008	0.788	0.674	<0.001***
Pog-Pog’	0.002	0.455	0.047	0.004**	0.026	0.030*	0.042	0.006**	0.025	0.033*	0.075	<0.001***	0.073	0.001**
Gn-Gn’	0.058	<0.001***	0.032	0.016*	0.060	0.001**	0.089	<0.001***	0.130	<0.001***	0.102	<0.001***	0.153	<0.001***
Me-Me’	0.002	0.435	0.106	<0.001***	0.013	0.110	0.106	<0.001***	0.006	0.206	0.119	<0.001***	0.119	<0.001***

*P < 0.05. **P < 0.01. ***P < 0.001. Numbers in bold represent the highest result of interaction between two or all independent variables.

There was a weak association between basal symphyseal thickness (Pog-Pog”) and the inclination of the basal symphysis (AL1Me.MP). However, sagittal position (V-Gn) interacted with the sagittal and vertical patterns, and explained almost 65% of the variability, in combination with the vertical pattern. The vertical position (H-Gn) was influenced by sex and vertical pattern, and explained 29% of vertical position variation.

The soft tissue position (G’perp-Pog’) was mainly influenced by sagittal and vertical patterns, providing the main explanation for all variations (*R* ^*2*^= 0.673). Soft tissue thickness was weakly influenced by sex and age (Pog-Pog’, *R* ^*2*^= 0.07; Gn-Gn’, *R* ^*2*^ ^^= 0.102; Me-Me’, *R* ^*2*^ ^^= 0.119).

## DISCUSSION

Knowledge of the factors associated with the morphology of mandibular symphysis and adjacent tissues can be useful for planning genioplasty, which can be performed as an isolated procedure or as part of a more complex orthognathic surgery. To our knowledge, no previous study has explored the concept of symphysis morphology, including the surrounding tissues, using multiple regression analysis. This point allows to clarify the isolated and combined factors that can influence the morphology of the mandibular symphysis. This understanding may have a direct impact on clinical procedures, due to the possibility of reshaping only one type of tissue (skeletal or soft) or to modify both, considering sex and age. In general, the present study showed that the symphysis and soft tissue chin is a complex anatomical site influenced mostly by sagittal and vertical patterns, and complemented by sex and age, impacting differently on the alveolar, basal and soft tissue portions.

The present adult sample was well distributed in terms of sex and skeletal patterns and ranged from a balanced facial skeletal pattern to extreme discrepancies in the vertical and sagittal pattern. Such a miscellaneous sample is commonly researched to capture symphysis and surrounding tissue variations.^4^ ^,^ ^14^ ^,^ ^15^

Previous studies^11^ ^,^ ^16^ ^,^ ^18^ have demonstrated that alveolar symphyseal inclination and thickness are influenced by sagittal and vertical skeletal patterns. These findings were also confirmed by the present results. For this reason, it was expected that vertical pattern could have a strong correlation with symphysis thickness, a fact not confirmed by the present results (r = 0.166). Another interesting data was that alveolar thickness maintained stable with aging. This study used a sample with patients ranging from 16 to 63 years old (most of them young adults). The older group showed a mean reduction of only 0.5 mm in the alveolar symphysis, and it was not considered statistically significant. Pre-surgical treatment in preparation for orthognathic surgery is a major clinical concern, especially in patients with Class III skeletal malocclusion and a hyperdivergent pattern. Forward orthodontic incisor inclination in a thin alveolar width could cause iatrogenic damage, such as dehiscence and fenestration, and could be associated with the development of gingival recession,^5^ ^,^ ^23^ although a recent cohort study concluded that symphyseal morphology is not a risk factor for the occurrence of gingival recession.^24^

The position of the basal symphysis was naturally influenced by the sagittal and vertical skeletal patterns^11^ ^,^ ^16^ ^,^ ^18^. Gomez et al.^17^ found that symphysis morphology measurements do not show significant relationships between skeletal pattern or between vertical patterns independently, but relationships are found when both parameters are associated, such as basal symphyseal inclination. The present results also showed an association of sagittal and vertical skeletal patterns in basal symphyseal inclination, but weak association with thickness. Basal symphyseal inclination was similar in skeletal Classes I and III, and in low and high mandibular plane angles. The basal lingual inclination detected in another study^12^ on Class III mandibular symphysis was not corroborated by the present findings. Their study used a different methodology, in which basal inclination was influenced by mandibular incisor inclination. On the other hand, basal symphyseal thickness was not influenced by the studied variables and seemed to vary individually due to strong genetic determination. This data must be well explored in future studies, searching for specific genes that may contribute for the symphysis morphology.

In general, male anatomical dimensions and symphyseal linear measures are larger than those of females. Thickness, anteroposterior, and vertical positions, and various soft tissue measurements were greater in males, which is in accordance with previous studies.^4^ ^,^ ^18^ ^,^ ^20^ ^,^ ^23^ However, no sex difference was found in the soft tissue chin projection, as previously demonstrated in a sample of a balanced face,^11^ and could be explained by Enlow’s hypothesis of growth equivalence.^24^ Symphyseal height is, on average, 10% greater in males than in females, in both balanced and unbalanced faces, as shown by the present sample, and this result is in agreement with Gomez et al.^17^ This feature also includes the height of the incisor crown. 

Chin soft tissue was influenced by sex and age, even in adulthood. The sagittal and vertical patterns can predict only the position, contrasting with previous studies on soft tissue chin thickness that have shown the influence of various patterns of mandibular divergence and sex.^4^ ^,^ ^14^ When age groups are compared, the present results corroborate findings that soft tissue thickness could increase with aging. This fact has already been observed longitudinally in individuals during growth.^18^ A previous study showed that the soft tissue increased nearly 2mm between the ages of 6 and 18 years old.^25^ The present results showed that Gn’ thickness increased 1.7mm during adulthood, which was lower than in another study^19^ that found a 3.7-mm increase over a 40-year period, considering from late adolescence to late adulthood. Age was also related to shortness of vertical basal symphysis position in the present sample. One possible explanation for this finding could be physiological tooth wear throughout life, and its consequence for the vertical dimension of the face.^27^ Thus, longitudinal studies in adults should be encouraged to detect individual, sex, sagittal and vertical pattern variations over time, as age seems to be an influencing factor for both the vertical position of the symphysis and soft tissue thickness. This information could help facial surgeons on planning surgical interventions, and also inform surgical patients about the physiological changes throughout life.

This study has limitation regarding the cross-sectional design. Because the study was not based on a longitudinal data set, the association established needs to be carefully interpreted. However, the sample groups were allocated with comparable distribution, and also statistical adjustment contributed towards minimizing bias and confounding effects. In addition, the 2-D cephalometric measurements confirmed satisfactory reproducibility. Although 3-D evaluation is the current approach in skeletal researches, it is not recommended for all patients in orthodontics.^28^ ^,^ ^29^ For ethical reasons and ALARA principle, the 3-D tomographic images must balance the risks and benefits, especially in patient with no treatment need (normal occlusion).^28^ ^,^ ^29^

**Figure 3:**
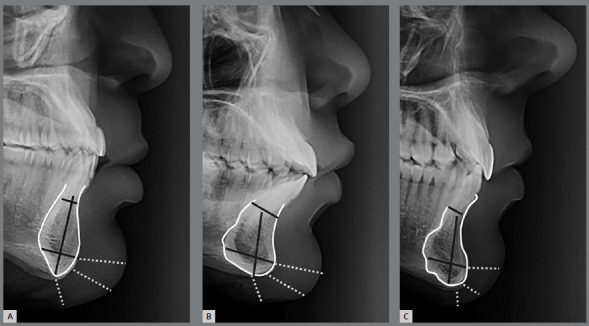
Variation in the morphology and dimension of the symphysis and surrounding tissues. A) Tall and narrow; B) Short and wide; C) Wide in the basal portion and narrow in the alveolar. No prediction of soft tissue chin can be estimated from the skeletal dimensions.

Genioplasty can be designed to increase or reduce chin size or to straighten an asymmetrical chin. Although the response of soft tissues is similar to bone movement, genioplasty should be performed with discretion and individually (Fig 3). Two of the influence factors, such as age and sex, cannot be manipulated clinically, and together have influence on symphysis height and soft tissues thickness. The aging influence in each gender must be useful on the treatment planning of genioplasty. Older patients must have compensations about the probable shortening of the vertical height of the chin. The clinician must plan which tissue must be transformed (skeletal, soft tissue or both) in order to reach the best result on vertical position of the chin, also considering relapse of soft tissues in older patients. Additionally, since the morphology and position of the soft tissues can be altered by aging, it is essential to future studies to investigate predictive criteria for changes from hard to soft tissues, and apply this information not only after surgery but also in long term periods. The present study reinforces that the thickness of soft tissue chin is not necessarily related to the size of the underling skeletal pattern and, in addition, the influence of sex and age cannot be disregarded.

## CONCLUSIONS

This study concludes that the morphology of symphysis and surrounding tissues are influenced by sex, age, and sagittal and vertical patterns variables, which acts differently in its alveolar, basal, and soft tissue portions. Sagittal and vertical patterns had the strongest association on alveolar symphysis inclination and soft tissue horizontal position. Sex and age can influence the basal symphysis position and soft tissues thickness. The varied morphologies corroborate the need for a strictly individualized planning of genioplasty.

## References

[1] Sherwood RJ, Hlusko LJ, Duren DL, Emch VC, Walker A (2005). Mandibular symphysis of large-bodied hominoids. Hum Biol.

[2] Yu Q, Pan X, Ji G, Shen G (2009). The association between lower incisal inclination and morphology of the supporting alveolar bone a cone-beam CT study. Int J Oral Sci.

[3] Nojima K, Nakakawaji K, Sakamoto T, Isshiki Y (1998). Relationships between mandibular symphysis morphology and lower incisor inclination in skeletal class III malocclusion requiring orthognathic surgery. Bull Tokyo Dent Coll.

[4] Gomez Y, Zamora N, Tarazona B, Bellot-Arcís C, Paredes-Gallardo V (2017). Cross-sectional human study of soft tissue chin (STC) thickness in adult patients in relation to sex, facial pattern and skeletal class. J Craniomaxillofac Surg.

[5] Handelman CS (1996). The anterior alveolus its importance in limiting orthodontic treatment and its influence on the occurrence of iatrogenic sequelae. Angle Orthod.

[6] Herzog C, Konstantonis D, Konstantoni N, Eliades T (2017). Arch-width changes in extraction vs nonextraction treatments in matched Class I borderline malocclusions. Am J Orthod Dentofacial Orthop.

[7] Aziz T, Flores-Mir C (2011). A systematic review of the association between appliance-induced labial movement of mandibular incisors and gingival recession. Aust Orthod J.

[8] Skieller V, Björk A, Linde-Hansen T (1984). Prediction of mandibular growth rotation evaluated from a longitudinal implant sample. Am J Orthod.

[9] Misch CM, Misch CE, Resnik RR, Ismail YH (1992). Reconstruction of maxillary alveolar defects with mandibular symphysis grafts for dental implants: a preliminary procedural report. Int J Oral Maxillofac Implants.

[10] Hoenig JF (2007). Sliding osteotomy genioplasty for facial aesthetic balance: 10 years of experience. Aesthetic Plast Surg.

[11] Arruda KEM Valladares Neto J, Almeida G de A (2012). Assessment of the mandibular symphysis of caucasian brazilian adults with well-balanced faces and normal occlusion: the influence of gender and facial type. Dental Press J Orthod.

[12] Al-Khateeb SN, Al Maaitah EF, Abu Alhaija ES, Badran SA (2014). Mandibular symphysis morphology and dimensions in different anteroposterior jaw relationships. Angle Orthod.

[13] Gütermann C, Peltomäki T, Markic G, Hänggi M, Schätzle M, Signorelli L (2014). The inclination of mandibular incisors revisited. Angle Orthod.

[14] Macari AT, Hanna AE (2014). Comparisons of soft tissue chin thickness in adult patients with various mandibular divergence patterns. Angle Orthod.

[15] Khan MY, Kishore MS, Bukhari SA, Rachala MR, Sashidhar NR (2016). Alveolar and Skeletal Chin Dimensions Associated with Lower Facial Height Among Different Divergent Patterns. J Clin Diagn Res.

[16] Qu X, Liu Z, Wang Y, Fang Y, Du M, He H (2017). Dentofacial traits in association with lower incisor alveolar cancellous bone thickness a multiple regression analysis. Angle Orthod.

[17] Gómez Y, García-Sanz V, Zamora N, Tarazona B, Bellot-Arcís C, Langsjoen E (2018). Associations between mandibular symphysis form and craniofacial structures. Oral Radiol.

[18] Nanda RS, Meng H, Kapila S, Goothuis J (1990). Growth changes in the soft tissue facial profile. Angle Orthod.

[19] Buschang PH, Julien K, Sachdeva R, Demirjian A (1992). Childhood and pubertal growth changes of the human symphysis. Angle Orthod.

[20] Pecora NG, Baccetti T, McNamara JA (2008). The aging craniofacial complex a longitudinal cephalometric study from late adolescence to late adulthood. Am J Orthod Dentofacial Orthop.

[21] von Elm E, Altman DG, Egger M, Pocock SJ, Gøtzsche PC, Vandenbroucke JP (2007). The strengthening the reporting of observational studies in epidemiology (STROBE) statement guidelines for reporting observational studies. PLoS Med.

[22] Faul F, Erdfelder E, Lang AG, Buchner A (2007). G*Power 3 a flexible statistical power analysis program for the social, behavioral, and biomedical sciences. Behav Res Methods.

[23] Choi YJ, Chung CJ, Kim KH (2015). Periodontal consequences of mandibular incisor proclination during presurgical orthodontic treatment in Class III malocclusion patients. Angle Orthod.

[24] Mazurova K, Kopp J-B, Renkema AM, Pandis N, Katsaros C, Fudalej OS (2018). Gingival recession in mandibular incisors and symphysis morphology-a retrospective cohort study. Eur J Orthod.

[25] Enlow DH, Moyers RE, Hunter WS, McNamara JA (1969). A procedure for the analysis of intrinsic facial form and growth An equivalent-balance concept. Am J Orthod.

[26] Bergman R, Waschak J, Borzabadi-Farahani A, Murphy N (2014). Longitudinal study of cephalometric soft tissue profile traits between the ages of 6 and 18 years. Angle Orthod.

[27] Crothers A, Sandham A (1994). Vertical height differences in subjects with severe dental wear. Eur J Orthod.

[28] American Academy of Oral and Maxillofacial Radiology (2013). Clinical recommendations regarding use of cone beam computed tomography in orthodontics Position statement by the American Academy of Oral and Maxillofacial Radiology. Oral Surg Oral Med Oral Pathol Oral Radiol.

[29] European Commission (2012). Radiation protection: no 172, cone beam CT for dental and maxillofacial radiology (evidence-based guidelines).

